# Vision-Guided MPC for Robotic Path Following Using Learned Memory-Augmented Model

**DOI:** 10.3389/frobt.2021.688275

**Published:** 2021-07-26

**Authors:** Alireza Rastegarpanah, Jamie Hathaway, Rustam Stolkin

**Affiliations:** ^1^ Department of Metallurgy and Materials Science, University of Birmingham, Birmingham, United Kingdom; ^2^ The Faraday Institution, Harwell Science and Innovation Campus, Didcot, United Kngdom

**Keywords:** machine learning, dynamic modeling, electric vehicles, cutting, predictive control, vision

## Abstract

The control of the interaction between the robot and environment, following a predefined geometric surface path with high accuracy, is a fundamental problem for contact-rich tasks such as machining, polishing, or grinding. Flexible path-following control presents numerous applications in emerging industry fields such as disassembly and recycling, where the control system must adapt to a range of dissimilar object classes, where the properties of the environment are uncertain. We present an end-to-end framework for trajectory-independent robotic path following for contact-rich tasks in the presence of parametric uncertainties. We formulate a combination of model predictive control with image-based path planning and real-time visual feedback, based on a learned state-space dynamic model. For modeling the dynamics of the robot-environment system during contact, we introduce the application of the differentiable neural computer, a type of memory augmented neural network (MANN). Although MANNs have been as yet unexplored in a control context, we demonstrate a reduction in RMS error of ∼21.0% compared with an equivalent Long Short-Term Memory (LSTM) architecture. Our framework was validated in simulation, demonstrating the ability to generalize to materials previously unseen in the training dataset.

## 1 Introduction

Modern robots equipped with force and torque sensing capabilities offer a flexible platform to expedite a range of manual and repetitive tasks through contact and interaction with their environment. For such applications as milling, grinding or polishing, one such capability is to define and track a desired trajectory or path along the surface of an object while applying a given force. This is done such that task progression and progression along the tool path is enabled, while modulating the contact force to avoid damage to the tool and maintain the workpiece quality. However, emerging applications for robotics impose ever more challenging requirements on the flexibility of task specification and the control system. This can be separated into two principal problems: the first, allowing tool paths to be defined expeditiously while allowing for the provision of prior knowledge; and the second, enabling a robot to follow a desired path on an object where there are parametric uncertainties regarding the material properties and path planning with respect to the object surface. Traditionally, progress in these areas has been pursued separately. Existing works either emphasize flexibility in path planning through advanced vision-based methods and novel sensor capabilities, or flexibility in the control framework through application of adaptive compliant controllers, model predictive control, or more recently, learning-based model predictive control. Emphasizing the successes of the latter, a combination of path planning, visual servoing and tactile feedback with model predictive control is proposed to address both problem areas simultaneously.

**FIGURE 1:**
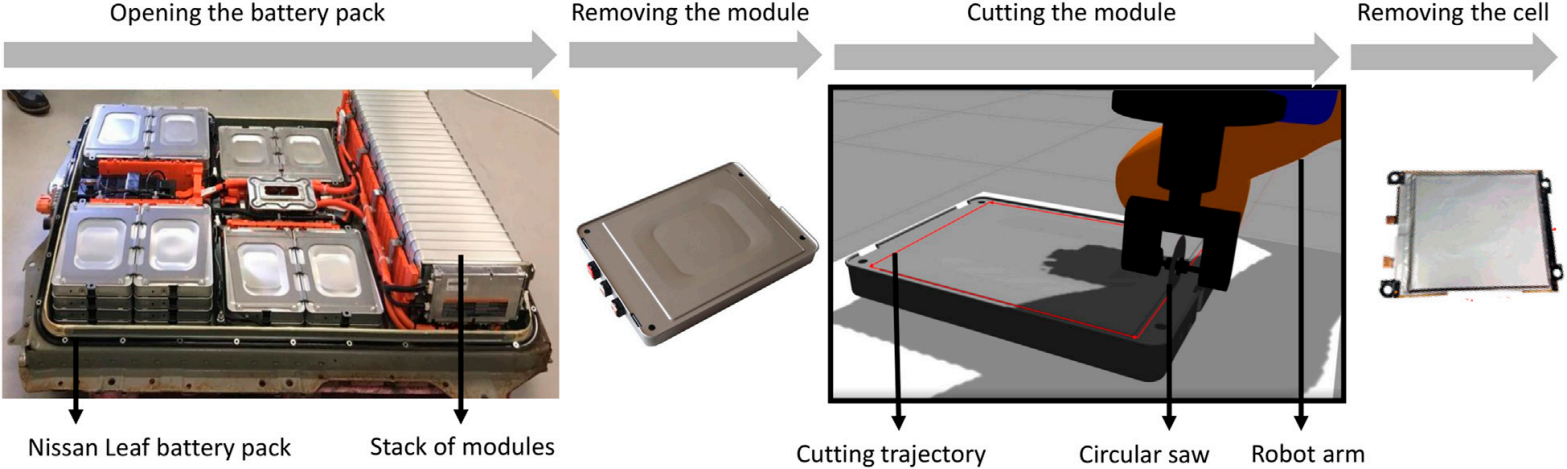
Schematic example of an industrial application of flexible path following control: extraction of battery cells by mechanical separation (cutting) of the module cover.

For path-following control, an exemplar application of industrial relevance is the emerging area of robotic disassembly of lithium-ion batteries (LIBs) (Figure 1). Accessing high-value components such as cells by milling and mechanical separation of the battery module casing presents a challenging problem due to lack of standardization of battery designs, leading to parts with differing geometries and materials. This results in the task specification and contact dynamics changing between tasks. For this reason, current LIB disassembly practices are manual and functionally rudimentary. Traditional control schemes for contact-rich tasks, such as impedance control, are limited due to the need to manually tune controller parameters (Siciliano and Khatib, 2008), which is impractical in this case. While adaptive schemes have been demonstrated to be robust to uncertainties in environment properties (Duan et al., 2018), they are typically limited by fixed assumptions being made about the contact dynamics, and define a greedy policy that has been noted to be slow to adapt to sudden changes in environment properties (Mitsioni et al., 2019).

In the presence of environment uncertainties, a further consideration for path following tasks is the ability to follow a predefined path in a trajectory-independent manner. That is, without prior prescription as to the precise trajectory, which requires accurate task planning, or process parameters, for example, the feed velocity in milling processes. This setup, known as the path following problem, has been explored in Faulwasser et al. (2017) based on model predictive control (MPC) for a collaborative robot, demonstrating the capability to follow a prescribed path in contact with the environment with automatic adjustments to the path velocity. In Matschek et al. (2017); Meng et al. (2020), this treatment was extended to allow explicit control of the contact forces to a setpoint. In Stemmler et al. (2019), predictive control was applied for dynamic feed-rate adjustment for control of force during CNC milling processes. However, all of these approaches depend on an explicit model-based formulation of MPC, which is still dependent on prior knowledge of the environment. For tasks such as milling, an analytical model-based framework quickly becomes intractable due to the complexity of the cutting dynamics, warranting detailed computational modeling approaches such as finite-element modeling (Meng et al., 2019) that are currently unsuitable for real-time deployment. Even simpler analytical models are highly dependent on the geometry of the cutting task, the process variables and the nature of the workpiece material (Potoc̆nik and Grabec, 2002; Stemmler et al., 2019).

With these limitations in mind, the introduction of learning-based methods has presented considerable advantages in flexibility. In Mathew et al. (2019) a feed-forward Gaussian mixture dynamics model was used to guide a variable impedance controller, showing the capability to adapt to a range of materials with variable stiffness, viscosity and surface friction. For learning-based MPC, similar works have focused on learning a dynamic model of the cutting process for a range of food articles (Lenz et al., 2015; Mitsioni et al., 2019). In these works, traditional tracking control approaches fail as the advancing reference window causes the tool to become embedded in the material, leading to continuously increasing contact forces that immobilize the cutting tool. However, for these approaches, emphasis must be given to the capability of the model to learn and accurately model the dynamics. The model must have sufficient representation power to learn a complex, potentially non-linear dynamics function while also generalizing to new data if the model is to be informative for a wide range of material classes. Recurrent architectures such as the LSTM are popular and well-known for their application to complex time-series problems, as well as in the context of robotics. However, it has been suggested (Chen et al., 2017; Mufti et al., 2019; Ming et al., 2020) that the LSTM is further outperformed when considering recurrent NN architectures coupled with external memory, or memory-augmented neural networks (MANNs). This is of particular interest for problems where long-term dependencies are present in the data. Furthermore, beyond the modeling aspect, it may be necessary to modify the reference path online to make the process robust to variations in the surface geometry from task to task. These factors include the contours of the part surface between differing designs, bending or deformation during machining, or even damage to the part sustained during or after its service life.

We provide an introduction of previous learning-based MPC approaches, before introducing the principle of memory-augmented neural networks. Finally, we summarize related literature incorporating the use of vision in the context of contact-rich tasks, such as milling or grinding. Thereafter, the main contributions of this work are summarised.

### 1.1 Learning-Based Model Predictive Control

Examples of explicit, model-based MPC-based control schemes are prevalent in the literature. An alternative is to use learning-based approaches, such as neural networks, to construct a dynamic model of the controlled system. An advantage of modeling the contact dynamics with neural networks is the ability to learn an arbitrary, potentially non-linear function describing the process state evolution, with the principal limitation then shifting to the requirement for training data. As part of an MPC framework, this represents a key distinction from similar approaches such as model-free reinforcement learning, where the policy must be learned directly by interaction with the system. This is crucial for the cutting application due to the destructive nature of the tasks being executed. Although this approach is not new (Potoc̆nik and Grabec, 2002), it has been the subject of continued and recent exploration due to its applicability to a wide range of tasks (Williams et al., 2017; Nagabandi et al., 2018; Ay et al., 2019; Chen et al., 2019; Mitsioni et al., 2019).

More closely related to our case study of the battery cutting application, Ay et al. (2019) explored optimization of the milling process feed velocity based on a support-vector machine dynamic model. In this case, improvements in both productivity and modeling accuracy were achieved over an existing empirical model-based method. A form of path following predictive control was developed in Lenz et al. (2015) based on a neural network model of the dynamics of cutting of food articles using a knife tool, showing the capability to generalize to a wide range of foods with different physical properties. In Mitsioni et al. (2019) this approach was reformulated for position-controlled robots with an improved network architecture. Although the strategy explored in these works is similar to the path following applications presented in Faulwasser et al. (2017); Meng et al. (2020), there is no mechanism for the provision of the desired cutting path. This is appropriate if both the precise cutting path and trajectory are irrelevant to the task, but is insufficient for cases where geometric or safety concerns mandate explicit control over the path, such as cutting of battery components. Examples of recent learning-based predictive path following studies are Yang et al. (2021) and Wu et al. (2021). Yang et al. (2021) consider a general path following control framework using a Gaussian process (GP) estimator to adapt to external disturbances. However, the paper is applied to the quadrotor which is a system with well-defined dynamics. Therefore, although non-parametric uncertain disturbance is considered, the method is not robust to system parametric uncertainty. Wu et al. (2021) propose a predictive tracking control method based on a neural network, robust to parametric uncertainties. However, the paper employs a simple RNN dynamic model; this could be improved upon by considering improved architectures such as the LSTM as addressed in Mitsioni et al. (2019). Further architectures could be considered; this will be discussed in Section 1.2.

Alternative approaches consider a combination of learning-based MPC with a learned policy, or approximate MPC, where the former is applied offline to train the latter for online deployment. Safavi et al. (2015) employed a combination of a multi-layer perceptron dynamic robot model and MPC to optimize the force feedback guiding the point-to-point movement of a manipulator assisted by a human operator. Bonzanini et al. (2021) proposed an approximate multi-stage MPC framework accounting for time-varying uncertainties and model-plant mismatch. However, this approach in principle still depends on a nominal (known) model of the dynamics. Model-based reinforcement learning (RL) was applied in Nagabandi et al. (2018) in simulation to learn gaits for point-to-point movement and path following. In this case, an MPC framework with a learned dynamics model was trained off-task using random inputs, using this to train a model-free RL policy. While these approaches are advantageous as the training sample requirements of RL are reduced and real-time action optimization does not have to be considered, the learned policy is no longer independent of the task objectives. Therefore, it is unclear whether the desired paths may be adjusted to compensate for path planning uncertainties in real-time. Beyond learning-based predictive control, in Tutsoy et al. (2018) a model-free reference tracking control architecture was presented. Although the method is robust to both parametric and non-parametric system uncertainties and does not require training, the method is only applicable to systems with linear dynamics.

### 1.2 Memory Augmented Neural Networks

Previously, many learning-based methods incorporated into an MPC framework have used feed-forward neural networks or recurrent architectures such as the LSTM. In recent years, however, it has been demonstrated for a range of applications such as time series forecasting, graph traversal and reinforcement learning, that a class of neural networks augmented with external memory, or memory-augmented neural network (MANN) have been demonstrated to outperform traditional recurrent neural networks and their extensions, due to their ability to account better for the long-term behavior of the modeled system, which presents compelling advantages for the control application.

We consider the differentiable neural computer (DNC), introduced in 2016 by DeepMind (Graves et al., 2016), as an extension of the earlier neural Turing machine (NTM) architecture—a type of MANN. We summarize the principle of operation of the DNC in brief, however, an exhaustive description is detailed in Graves et al. (2016). The DNC architecture comprises a controller network, typically a recurrent neural network such as an LSTM (as chosen in the original study), and a memory unit, consisting of a memory matrix of size N×W, where *N* is the number of memory locations and *W* is the word size. When the network is inferred at time step *t*, the input to the controller network is formed by an augmented input matrix comprising the external network input concatenated with *R* read vectors $r_{t-1}\in {\mathbb{R}}^{W}$ from *R* read heads, while the output consists of an output vector $v_{t}$ and an interface vector $\xi_{t}$. The vector $\xi_{t}$ is used to update the internal state of the memory matrix. Interaction of the controller with specific regions of the memory matrix is mediated through multiple attention mechanisms which are end-to-end differentiable. For the read heads, content-based addressing defines a weighting distribution over the memory locations based on the cosine similarity to a *W* element read key vector contained in $\xi_{t}$, while an N×N temporal link matrix stores associations between consecutively accessed memory locations. After each update, the final output to the DNC $y_{t}\in {\mathbb{R}}^{Y}$ is the sum of $v_{t}$ and the weighted next set of read vectors $r_{t,r}$, with weighting matrix $W_{r}\in {\mathbb{R}}^{RW\times Y}$

$$y_{t}=v_{t}+W_{r}\left[\begin{array}{cccc} r_{t,1} & r_{t,2} & \cdots & r_{t,R} \end{array}\right]$$ (1)

The original study by Graves et al. (2016) demonstrated competencies in a wide range of problems, including natural language processing, question answering and navigation tasks. These findings are supported by a consensus in related literature, suggesting the DNC architecture is capable of outperforming simple recurrent neural networks (RNNs)/LSTMs, especially when considering data that has long-term dependencies. For example, most recently, stacked DNCs have been applied for analysis of electroencephalogram data in Ming et al. (2020). In this work, the DNC demonstrated best-in-class accuracy for mind load classification and reaction time inference when compared to LSTM recurrent models, 1D time series convolution, and a combination of LSTM with CNN-based latent feature extraction. More closely related to this work, Chen et al. (2017) employed a DNC with an LSTM controller in a reinforcement learning (RL) framework to learn a policy for navigation tasks. It was demonstrated that, while more prone to over-fitting due to the increased number of learnable parameters associated with the memory attention mechanisms, when proper regularization was applied to the network to counter this effect, the DNC demonstrated significantly improved generalizing capability over the LSTM. In Mufti et al. (2019), a combination of an ANN RL agent and a DNC environment model were combined to solve path and grid-based navigation tasks. The method achieved superior accuracy compared with the equivalent LSTM for learning a first principles environment model, however, the proposed framework has only been validated for simple case studies. Hence the relevance of this method to industrial applications remains to be explored.

The incorporation of a separately addressable memory allows the DNC to encapsulate long term dependencies on an order far exceeding that of traditional RNN/LSTM based models. This is especially useful in a control application where the feedback sampling rate of a closed-loop robotic control system can be on the order of hundreds to thousands of times per second. However, in spite of these advantages, literature pertaining to MANNs and their applications remains sparse, and to our best current knowledge, unstudied in a robot control context. Concerning DNCs in particular, a principal issue is that the architecture suffers from high computational complexity due to the network’s attention mechanisms (Rae et al., 2016), which presents a challenge for the control application to ensure the system is computationally tractable.

### 1.3 Vision-Based Feedback and Path Planning

Among other drawbacks, such as cost, autonomous identification of key components such as cells remains a challenging problem, with the most straightforward recourse to prior knowledge through the use of detailed CAD models, which may not be available for all designs, extensive vision datasets beyond conventional depth camera or image datasets—themselves underdeveloped for EV batteries, or operator experience. It is precisely these requirements that motivate a vision-based approach to path planning, as well as incorporation of real-time visual feedback to assist path following tasks along the surface of an unknown workpiece. Vision-based path planning approaches have been proven to be robust and effective at accomplishing and assisting a wide range of contact-rich tasks, but are mainly motivated by industry-specific applications. In Wang et al. (2020), the problem of autonomous planning of a grinding tool path over weld seams was addressed based on local surface reconstruction using point cloud data. A framework leveraging flawed point cloud data with holes, noise and discontinuities was proposed in Barnfather and Abram (2018) for compensation of dimensional errors in machining processes. Similar approaches are presented in Li et al. (2018) and Zhen et al. (2019) for the grinding of an unknown workpiece. Although these previous works have incorporated advanced and autonomous methods for path planning based on visual feedback, many of these schemes are inappropriate for more generic applications such as robotic disassembly, due to the requirement for some degree of prior knowledge regarding the task specification, workpiece geometry and internals.

Addressing the application-specific nature of the path planning has been considered in Amersdorfer et al. (2020). In this case, vision-guided control using depth sensors and image-based path planning was considered over free-form surfaces for machining tasks, integrating this with a combination of traditional impedance and direct force control schemes. However, all of the approaches considered are constrained by and validated with the aforementioned limitations of conventional control strategies for collaborative robots, or consider CNC tooling applications where there is emphasis on the selection of process parameters, such as feed rate, rather than optimization of the control system. Recently, the combination of a vision-guided serial manipulator with model predictive control was proposed in Fehr et al. (2020), using this to overcome limitations for accurate determination of the end-effector pose. Although the study considers explicitly the integration of path planning into the control framework, the MPC approach is still reliant on an explicit formulation of the task dynamics in contrast to the learning-based approaches detailed. Most recently, the use of MPC with a learned dynamic model based on environment location feedback was proposed in Sonker and Dutta (2021) for path tracking of a wheeled robot on uneven terrain, where the use of terrain elevation data was directly incorporated into the dynamic model to improve prediction accuracy.

### 1.4 Summary

To address the limitations of existing methods, a new method for robotic path following for contact-rich tasks is presented, based on the fusion of image-based path planning and vision with model predictive control. Considering the challenges of an explicit model-based formulation of MPC to construct a generalized task dynamics model, we formulate our MPC framework based on a learned state-space dynamic model. This work introduces the application of a memory-augmented neural network (MANN) in a control context, based on the differentiable neural computer (DNC), introduced by DeepMind in 2016. To the best knowledge of the authors, this is the first study investigating the application of a MANN in a control application.

The main contributions of this paper are:

• A proof-of-concept MPC implementation using a NN-based dynamic contact model, enabling trajectory-independent robotic path following in the presence of parametric uncertainties. We consider the additional constraint of low data collection requirements.
• An introduction of the use of MANNs in a control context, their technical challenges, and comparison with LSTM/simple RNN architectures.
• Integration of image-based path planning with MPC to enable paths incorporating prior knowledge to be rapidly defined for a range of object classes.
• Incorporation of visual feedback for contact-rich path following tasks, augmenting the adaptive behavior of the control framework; e.g. to compensate for deformation of the object surface or imperfections in path planning.

## 2 Methodology

**FIGURE 2:**
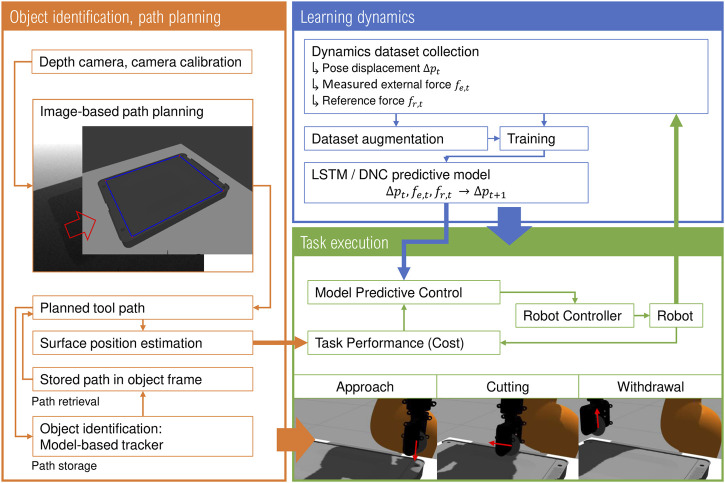
Graphical overview of the proposed method MPC-based path following framework for contact-rich tasks. The overall process is defined by the vision system, incorporating path planning, surface position estimation and storage and recovery of paths for different object classes, and learning of the tool-environment interaction model, which together guide the MPC approach.

We first introduce the vision-based path planning and feedback framework in Section 2.1, before bringing this into the broader context of our control framework in Section 2.2. Subsequently, we establish the structure of the environment for simulation of cutting trials and data collection for the learned dynamics model in Section 2.3. Finally, the aspects of data collection and model selection are considered in Section 2.4. A graphical overview of our proposed methodology is presented in Figure 2.

### 2.1 Vision-Based Path Planning and Feedback

Unlike the application presented in Mitsioni et al. (2019), it is important to maintain explicit control over the cutting path along the module surface due to the sensitivity of the components involved. In the cutting application presented, for example, the goal is to remove the cover of a typical battery module without damaging the sensitive cells within. However, this approach presents a problem when considering the large range of module designs in circulation. A path planning framework is hence proposed to deal with this limitation in two stages: in the first, the module pose is identified through the use of a model-based tracking algorithm (Trinh et al., 2018), given an approximate module pose is provided as input. In the second stage, point cloud data from an RGBD camera is used to directly define a tool path. To expedite the process of defining an appropriate tool path, we developed an image-based path planning framework. To define the path, the operator can define an arbitrary polygonal path of “nodes” in the image frame (u,v). These are converted to distorted image normalized coordinates (x′,y′) through the camera intrinsic calibration:

$$x^{\prime }=\frac{u-u_{0}}{p_{x}}\;y^{\prime }=\frac{v-v_{0}}{p_{y}}$$ (2)

based on the principal image point $\left(u_{0},v_{0}\right)$, and focal lengths $p_{x}$, $p_{y}$. Then, assuming radial distortion with a distortion coefficient $k_{du}$, the undistorted image point (x,y) may then be computed

$$x=x^{\prime }\left(1+k_{du}r^{2}\right)\;y=y^{\prime }\left(1+k_{du}r^{2}\right)$$ (3)

$$r^{2}=x^{\prime 2}+y^{\prime 2}$$ (4)

Given the undistorted image point in meters (x,y), the measured depth, pCn,z, of the point in the camera frame C may be used to obtain the components of each path vertex pCn using the projection

pCn,x=pCn,zx pCn,y=pCn,zy (5)

Finally, the obtained cutting path nodes are transformed into the robot base W and object frame O of reference as

pWn=TWCpCn (6)

**FIGURE 3:**
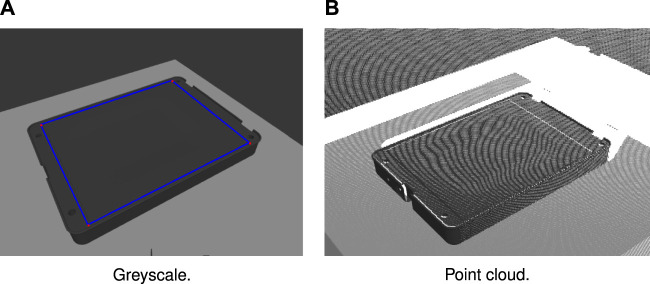
Example of image-based path planning, with greyscale image of the workpiece **(A)** with planned path defined, and point cloud generated from RGBD image **(B)** from which the camera frame path is inferred.

pOn=TOWpWn (7)

where TOW is the base frame matrix representation of the object pose estimated by the tracker. TWC is the camera frame matrix representation of the base frame pose, dependent on the camera extrinsic calibration and manipulator kinematics. By the inverse transformation to (Eq. 7), the base frame path may be recovered for any given object pose without having to redefine the original path. The path planning process is demonstrated in Figure 3, showing the raw point cloud data and visual representation of a schematic camera frame path.

However, the use of depth camera data typically comes with a number of caveats due to measurement imperfections. Most commonly, the collected data is noisy and suffers from holes in regions with reflections or shadowing. To improve the robustness of the vision system to noise, the raw depth image is first preprocessed using a Gaussian low-pass filter. Filling of holes is considered beyond the scope of this paper, however it is noted that a number of schemes exist for filling of holes and removal of discontinuities for point cloud imaging, and we assume the lighting conditions of the environment can be controlled to reduce the effect of the workpiece reflectivity. During the task execution, it is desirable to refine the reference path online by estimation of the position of the object surface in real-time along the path. This is achieved by considering the planned path in the camera frame C by the inverse transformation to (Eq. 6). At each time, the current position of the tool center point is projected onto the cutting path to obtain a sample point pCs. The estimate of the module surface position is then computed by considering the Gaussian weighted average of the *K* points in a given radius *r* obtained using a brute-force search over the point cloud (Rusu and Cousins, 2011). This effectively constitutes a second low-pass filter in 3D across the local neighborhood of pCs. Accordingly, the workpiece surface position estimate is then defined as

pCe={∑k=0K1(2πr)3  exp(−(pCk−pCs)22r)pCkif K>0pCsotherwise (8)

This has the advantage of flexibility, such that the surface position estimate is based on the *K* nearest neighbor sample points if feedback is available around the sample point, while deferring to the sample point, which lies on the original planned path, otherwise.

### 2.2 Control System Architecture

To enable tracking of a predefined trajectory, the baseline control system employs a variant of Cartesian inverse damping control (Siciliano and Khatib, 2008) as introduced in Mitsioni et al. (2019).

$$u=K_{a}\left(f_{e}-f_{r}\right)$$ (9)

where *u* is the velocity control input, $K_{a}$ is the compliance matrix, $f_{e}$ is the measured external torque, and $f_{r}$ is the reference force. All quantities are referred to in the robot base frame W. By choosing

$$f_{r}=K_{a}^{-1}\left(K_{p}e-{\dot{p}}_{d}\right)$$ (10)

and noting the velocity control input computed from (Eq. 9), we arrive at the control law

$$u={\dot{p}}_{d}+K_{a}\left(f_{e}-K_{a}^{-1}K_{p}e\right)$$ (11)

where ${\dot{p}}_{d}$ is the desired velocity, $K_{p}$ is the stiffness matrix. This can be thought of as a form of admittance control with effective stiffness and damping $K=K_{a}^{-1}K_{p}$, $B=K_{a}^{-1}$. Considering the case of a position/velocity controlled robot under the assumption the robot dynamics are largely decoupled by the low-level controllers, $\dot{p}=u$ (Siciliano and Khatib, 2008), this results in the closed-loop behavior

$$K_{a}^{-1}\dot{e}-K_{a}^{-1}K_{p}e=f_{e}$$ (12)

This represents the desired dynamic behavior in the special case of free space motion$f_{e}=0$. In the limit of infinite time, the position error converges to zero; the stiffness in this case only affects the transient behavior. In contact with the environment, the tool is offset from the desired trajectory proportional to the measured external wrench. Hence, the robot behaves as a mechanical admittance in response to an external force imposed by the environment. Unfortunately, for best performance, it is necessary to correctly tune the controller stiffness and compliance gains, and the selection of gains for one material or task may prove to be inappropriate for other materials. This presents a problem when considering the wide range of battery designs used in commercial EV batteries, where each design may have different material properties, with differing surface geometries, resulting in the dynamics changing from task to task.

Under the MPC approach, the path-following task can be reformulated as a discrete-time optimal control problem (OCP) for the current time *t*:

$$f_{r,t}^{\text{*}}=\underset{f_{r,t}}{\text{arg}\;\;\text{min}}\sum_{i=0}^{H}J\left(x_{t+i},f_{r,t+i}\right)$$ (13)

with the objective of minimizing a cost function *J* over a given horizon *H*. The cost function is a chosen metric evaluating the performance of the controller at accomplishing a desired task, as a function of a state vector $x_{t}$ and action at each time-step. In this case, we consider the reference force, $f_{r,t}$, as the action taken at each time-step. Solving the optimization problem in (Eq. 13), an optimal set of actions

$$f_{r,t}^{\text{*}}=\left\{f_{r,t}^{\text{*}},f_{r,t+1}^{\text{*}},\ldots ,f_{r,t+H}^{\text{*}}\right\}$$ (14)

resulting in the minimum cost may be generated. By considering not only the immediate influence of the action on the state and its convergence to a desired reference, but the long-term effects up to the horizon, this defines a proactive, rather than greedy policy. However, as the optimization problem must be solved under the constraints of a dynamic model of the system behavior, this implies the need for an accurate system model. Obtaining such a model directly is generally not possible in the presence of parametric uncertainties during contact with the environment. Therefore, using learning-based model predictive control, we aim to learn a discrete-time state-space representation of the task dynamics as

$$x_{t+1}=F\left(x_{t},f_{r,t}\right)$$ (15)

Here $F\left(x_{t},f_{r,t}\right)$ is a potentially non-linear target function describing the system dynamics. Based on the intuition of the state-space representation of the dynamics for linear systems and findings in Mathew et al. (2019), we incorporate the action ($f_{r,t}$) taken at each time step into the learned model.

As the condition of zero angular velocity parallel to the cutting path and normal to the surface forms the natural constraints of the cutting problem, and the orientation of the cutter perpendicular to these directions is fixed and relatively unimportant (due to the angular symmetry of the cutting tool) the orientation component of the cutting dynamics is neglected in the MPC framework for simplicity and to reduce the dimensionality of the underlying OCP. As shown in Figure 2, a contact-rich task such as cutting may be separated into three principal stages, the “approach” phase, the “cutting” phase and the “withdrawal” phase. In the approach phase, the cutter is positioned at a fixed location above the first cutting node and driven into the object surface. In the cutting phase, the cutter is moved along the cutting path through the material, separating it along the cutting path. Finally, in the withdrawal stage, the cutter is pulled from the object surface into free space and repositioned to the next node, parallel to the new cutting path. We hence construct our MPC approach with the objective of minimizing the cost function:

∑i=0HJ(pt+i,fr,t+i;pe,t+i,pn,1,pn,2)=∑i=0Hwslice||(pWn,2−pt+i)⋅c||+∑i=0Hwdev(pt+i−pWe,t+i)2+∑i=0Hwf(fr,t+i)2 (16)

$$c=\frac{p_{n,2}-p_{n,1}}{\left|\left|p_{n,2}-p_{n,1}\right|\right|}$$ (17)

with the current position of the end-effector $p_{t}$ and the path segment start pWn,1 and endpoint pWn,2. Note ||⋅|| refers to the ${\text{L}}^{2}$ norm. The cost function comprises three weighted components. Here, the first contribution consists of a slicing term weighted by $w_{slice}$, which drives the cutter forward toward the path endpoint pWn,2. The second, deviation term, with weighting $w_{dev}$, drives the cutter position toward the module surface position, and penalizes deviations from the desired path. A third term weighted by $w_{f}$ is included to encourage minimum effort ($f_{r,t+i}$) solutions and as a soft constraint to modulate the contact forces. For the deviation term, we introduce the surface position estimate pWe,t+i. However, the surface position is only estimated for the current real time *t*, i.e. pWe,t. As the position displacements at each time step are expected to be small, and to minimize computational overhead, the surface is assumed to be locally planar, and hence for each prediction the initial surface position estimate is displaced parallel to the cutting path as

$$p_{e,t+i}=p_{e,t}+\left[\left(p_{t+i}-p_{e,t}\right)\cdot c\right]\cdot c$$ (18)

**FIGURE 4:**
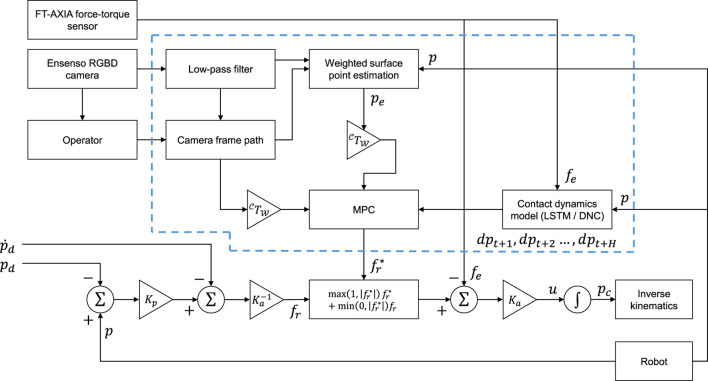
Block diagram for the proposed control system architecture based on vision-augmented MPC.

Typically, the desired action set is executed in a closed-loop fashion, by executing the first action $f_{r,t}^{\text{*}}$ and re-evaluating the new optimal action set. In general, it is difficult to solve (Eq. 13) for the optimal policy due to the inherent non-linearity of deep neural network models, while the high computational complexity of the model presents a trade-off in the solution being computationally tractable. On the other hand, the forward prediction of the state is a so-called “embarrassingly parallel” task, which lends itself well to the random forward shooting method. We generate N=256 random action sequences, from which the dynamic model is recursively inferred at each time step to generate a roll-out trajectory up to the MPC horizon. For each roll-out, the cost function is calculated, and the first action in the action sequence resulting in the lowest cost function is executed. The action sequences, in this case, are randomly sampled from a normal distribution $N\left(\mu_{action},{\text{Σ}}_{action}\right)$ at each time step, reflecting the measured action distribution of the training data. For this work, a horizon of H=10 time steps (0.2 s) is employed. The procedure for computing the action at each time step based on MPC with vision feedback is summarized in Algorithm 1. The overall control system architecture is represented graphically in the block diagram in Figure 4.

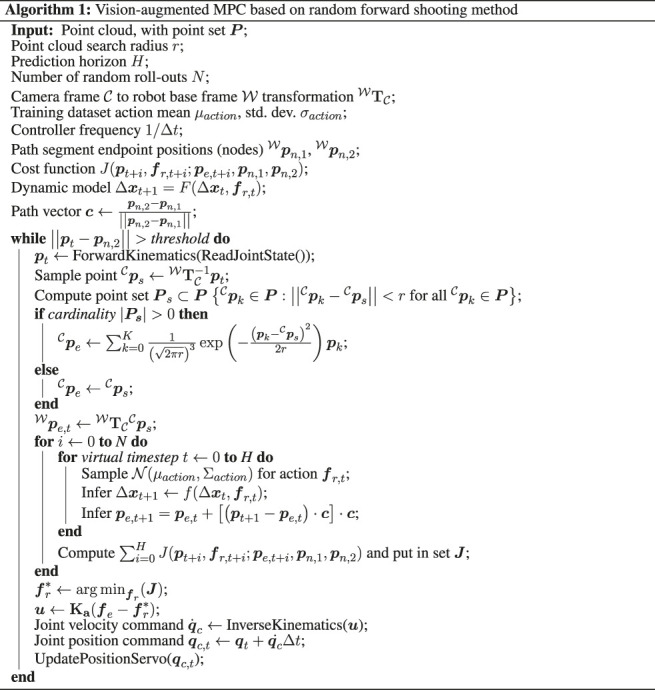

### 2.3 Cutting Simulation

**FIGURE 5:**
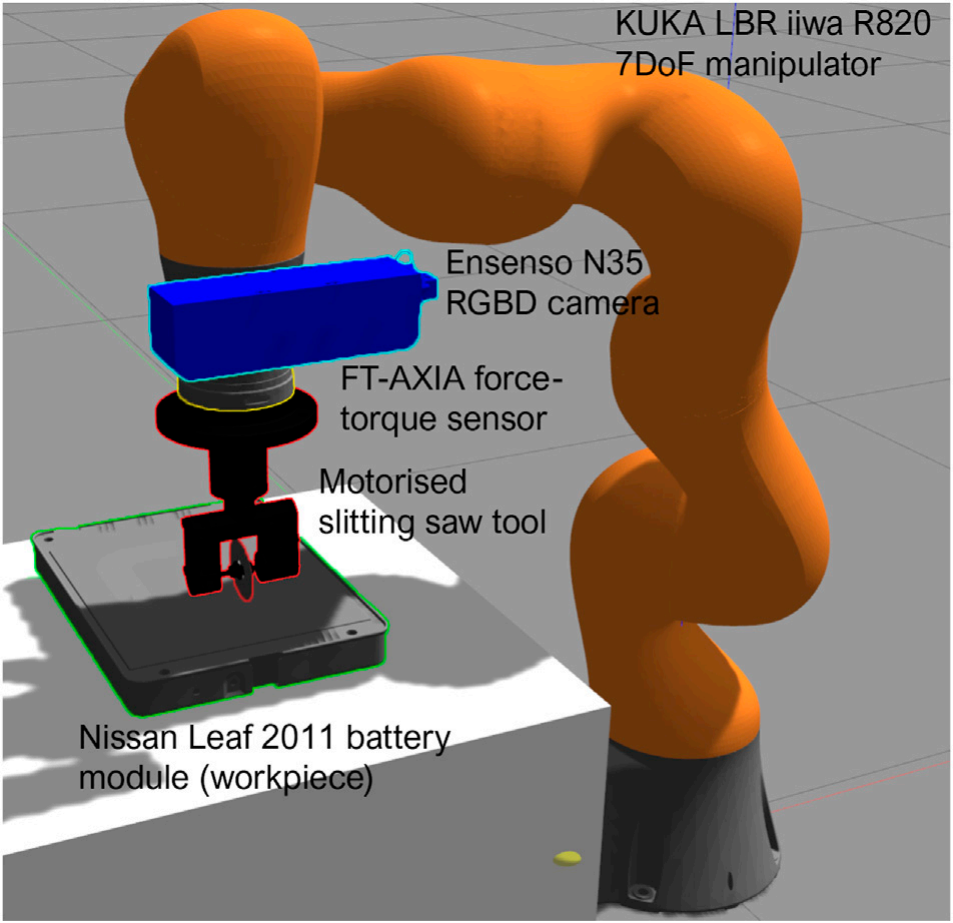
Simulation environment setup with KUKA LBR iiwa R820 collaborative robot equipped with slitting saw cutter tool, FT-AXIA force-torque sensor and Ensenso N35 RGBD camera, featuring an example Nissan Leaf 2011 battery module.

This work focuses on the implementation of the described algorithms using the KUKA iiwa LBR R820 collaborative robot in simulation based on the Robot Operating System (ROS) framework. This manipulator is equipped with a purpose-built circular saw cutting tool mounted onto an FT-AXIA force-torque sensor, and an Ensenso N35 RGBD camera. Development of the cutting simulation was carried out in Gazebo, with the environment shown in Figure 5. Fully accurate cutting simulation in real-time is technically difficult to implement. Few prior works have focused on developing infrastructure for modeling and simulation of the dynamics of cutting, with only basic simulations featuring deformation and separation of materials being developed for areas such as neurosurgery. Studies focusing on simulation of separation and cutting of material with a slitting saw implement are, for the most part, currently limited to detailed CAD/FEM computational models only (Meng et al., 2019). Hence, we adopt a simplified approach that aims to model roughly the cutting process but neglects many aspects of the dynamics of cutting. We treat the cutting problem as a surface-tracking problem with an elastically compliant material with constant (but potentially unknown) stiffness and isotropic surface friction. In this sense, the motivation is twofold:

• To validate the capabilities of the vision system for path planning and surface position estimation when integrated with the MPC framework.
• To validate the ability of the approach as a *proof-of-concept* to learn a generalized model of the task dynamics, which can then be applicable to a real-world cutting problem through transfer of the low-level knowledge obtained in simulation.

For navigation tasks required of the manipulator, such as positioning the end-effector at the starting position at each path node for each task, we employ the MoveIt ( et al., 2020) motion planning framework. The MoveIt framework encapsulates a number of robust motion planning algorithms, and is able to directly incorporate joint constraints, as well as knowledge of the environment and robot geometry to avoid collisions.

### 2.4 Data Preparation and Dynamics Model

Based on the path planning methodology described in Section 2.2, collection of training data was performed over ten manually generated polygonal surface paths (trials) over the surface of a Nissan Leaf 2011 battery module. The Leaf module is chosen for its predominantly planar geometry, while still having variation in the surface geometry as an application case study. We assume the case of an elastically compliant surface with isotropic, constant surface friction under parametric uncertainties. For most related works with an emphasis on development of adaptive compliant controllers, the most relevant parametric uncertainty of the robot-environment system under the set of assumptions considered is the surface stiffness. In this case, however, we also consider the influence of surface friction between the tool and workpiece. For each trial, surface properties were randomly chosen within a sample space summarized in Table 1 to introduce variation from task to task, so the model learns a generalized view of the contact dynamics. Although it has been suggested in principle that any interaction with the system is eventually sufficient for learning the dynamics (Williams et al., 2017), to generate a dataset most informative for the surface tracking tasks, we use the low-level damping controller to track a predefined fixed-rate trajectory on the workpiece surface. These desired position ($p_{d}$) trajectories were generated based on the defined surface path and the sliding rate chosen from Table 1 as

$${\ddot{p}}_{d}\left(t\right)=0,\;\;{\dot{p}}_{d}\left(t\right)=vc,\;\;p_{d}\left(t\right)=p_{n,1}+vtc$$ (19)

where *c* is the path unit vector, based on the node (i.e. path vertex) positions $p_{n,1}$, $p_{n,2}$ for each path segment as defined in (Eq. 17). Finally, for each task, the controller gains were then manually tuned to best accommodate the task.

**TABLE 1:** Sample space for surface properties selected for generation of dynamic model training data.

Property	—	Min	Max
Stiffness (Nm^−1^)	$k_{p}$	$10^{4}$	$10^{7}$
Tool-surface dyn. coeff. friction	μ	0.5	1
Sliding rate (m min^−1^)	*v*	0.3	1.8

Each data collection trial consists of a desired polygonal path composed of segments. For each path segment, bounded by $p_{n,1}$, $p_{n,2}$, virtual nodes are established at a displacement of d=0.05 m from the nodes parallel to the workpiece surface normal. Three sub-trajectories are generated from each pair of consecutive nodes as in (Eq. 19), corresponding to the approach, cutting and withdrawal stages respectively. While tracking the target trajectories, the data collected were the positions $p_{t}$ and measured external forces $f_{e,t}$, comprising the state vector $x_{t}=\left(p_{t},f_{e,t}\right)$. These are paired with the action consisting of the reference force $f_{r,t}$, calculated from the inverse damping control law in (Eq. 10). For the collection of validation data, a further two cutting trials were performed with an unseen set of parameters chosen from Table 1 as a bootstrap validation dataset. To generate the test dataset, a final trial is performed using the MPC framework with the trained model itself, over a manually specified polygonal path.

#### 2.4.1 Preprocessing and Input

A number of problems exist with the raw dataset collected for training. In the first instance, the time steps of the data collected are non-uniform, due to being synchronized between multiple sources of measurements. Moreover, due to the high computational complexity of the neural network-based MPC approach, it is unrealistic to infer the trained model after every state measurement. Thus, the data were subsampled to 50 Hz in order to generate a dataset with a consistent sampling rate. This consists of applying an FIR filter to the data to remove aliasing artifacts and decimation to the desired sampling rate. To accomplish decimation in real-time, a secondary buffer of the most recent state is kept, with full state updates being forwarded to the dynamics model after a “dead time” of 20 ms.

To generate the labeled datasets, the data were split into time windows of size T=40. In each window, T−H samples are used to initialize the state of the model, with the next *H* samples used as labels representing the ground truth future state. However, as the position of the desired cutting path is expected to vary from trial to trial, the controller predictions are expected to be translation-invariant, such that the model does not over-fit to specific positions. A robust and common method for handling this is to predict and train on the pose displacement $\text{Δ}p_{t}$ at each time step. Based on the state-space model formulation in (Eq. 15), we hence aim to learn a model of the form

$$\Delta x_{t+1}=F\left(\Delta x_{t},f_{r,t}\right)=f\left(X\right)$$ (20)

with $\text{Δ}x_{t}$ is the modified state $\left(\text{Δ}p_{t},f_{e,t}\right)$, *X* is the composite input vector $\left(\text{Δ}x_{t},f_{r,t}\right)$. Similarly, another key facet to ensure the robustness of the model is to prevent over-fitting to specific directions. Owing to the data collection schedule, the data collected were strongly distributed along specific directions, which could prevent the model from learning a more generalized view of the dynamics. To combat this, an augmented dataset was generated from copies of the original dataset. For each copy, a random rotation matrix $R_{aug}$ was generated, which is applied element-wise to each vector in the copied dataset as

$$X_{aug}=R_{aug}X$$ (21)

After generating the augmented input vectors $X_{aug}$, the original dataset is augmented with the copy. 20 copies were generated in total, resulting in an augmented dataset of 21 times the original size of the dataset. Finally, each training variable *X* was normalized to zero mean and a standard deviation of 1 as

$$X_{norm}=\frac{X-{\bar{X}}_{train}}{\sigma_{X,train}}$$ (22)

where $X_{norm}$ is the normalized value of the training variable, with mean and standard deviation of the variable in the training dataset ${\bar{X}}_{train}$, $\sigma_{X,train}$ respectively.

#### 2.4.2 Model Selection and Training

For learning the dynamics function in (Eq. 20), we consider two recurrent neural network architectures: the long-short term memory (LSTM) and the differentiable neural computer (DNC) as a type of MANN. For the DNC, it is necessary to choose the hyperparameters of the memory size *N* and word size *W*, forming the external N×W memory matrix. The contents of the memory are read by *R* read heads, each producing a *W* element vector concatenated with the external input *X* to the controller network. For this work, an LSTM is used as the controller network, in line with the original DNC study (Graves et al., 2016). Thus, at a glance, the DNC may be thought of as an LSTM with access to an external memory, which may be referred to as the model is repeatedly inferred. The remaining DNC hyperparameters were determined using a manual search. We take N=144, W=12 and R=4 for this work.

Both the LSTM and DNC controller networks are considered with the same underlying structure of a single recurrent layer with 32 hidden units, with two fully connected layers for input and output. The models were built and trained in Python using the TensorFlow library (Abadi et al., 2015). Training was carried out using the Adam (Kingma and Ba, 2014) optimiser on an NVIDIA GTX 1060 6 GB GPU, with a batch size of 256. The model was trained explicitly in an autoregressive fashion, such that the model is fed T−H state measurements, before predicting the future state *H* time steps forward. The final *H* predictions then are based solely on the previous predicted state and action taken at each time step. The model performance over *N* training samples was evaluated by mean squared error and mean absolute error as

$$\text{MSE}=\frac{1}{N}\overset{N}{\underset{t=0}{\sum }}\overset{H}{\underset{i=0}{\sum }}{\left({\hat{\Delta x}}_{t,t+i}-\Delta x_{t+i}\right)}^{2}$$ (23)

$$\text{MAE}=\frac{1}{N}\overset{N}{\underset{t=0}{\sum }}\overset{H}{\underset{i=0}{\sum }}\left\|{\hat{\Delta x}}_{t,t+i}-{\hat{\Delta x}}_{t+i}\right\|$$ (24)

where ${\hat{\Delta x}}_{t}$ denotes the model predicted value. Note that ${\hat{\Delta x}}_{t}$ is further indexed by *t*, as in principle the model predictions at each time-step are history-dependent.

## 3 Results and Discussion

### 3.1 Model Evaluation

**FIGURE 6:**
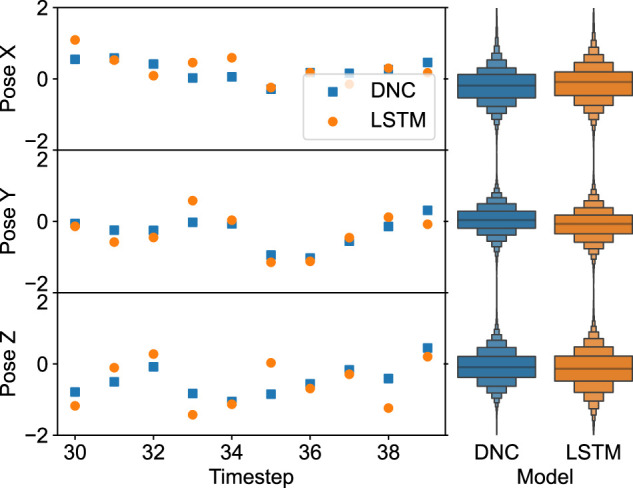
Example model residuals for pose displacement predictions over the horizon H = 10 using the LSTM (orange, light) and DNC (blue, dark) dynamics models, with the distribution of model residuals for each component shown as a boxen plot.

The predictions for both the LSTM and DNC dynamic models for the three components of position displacement over three sample windows for the validation dataset are shown. In this case, it is clear that both models are capable of predicting the future state of the system with reasonable accuracy, with the accumulation of error over autoregressive prediction having little effect on the predictions toward the end of the horizon. Comparing the two models, through the distribution of predictions and the RMS prediction error for each case, it is clear the DNC model predictions are more consistent. Indeed, for the example case presented in Figure 6, the majority of predictions from the DNC dynamic model are closer to the ground truth than the LSTM, suggesting the accuracy of the dynamics model benefits from the incorporation of the time history explicitly as part of the external memory.

To highlight this comparison, the RMS and mean absolute prediction errors are presented in Table 2. As a benchmark, the performance of a simple RNN model is considered for comparison, to place emphasis on the influence of the model’s ability to encapsulate long-term dependencies in the time series data. All three models are trained under the same conditions as outlined in Section 2.4. Comparison of these network architectures shows the RNN has the lowest accuracy of the three models. Since the simple RNN model does not have any mechanism to preserve state between non-consecutive time steps, this reduces the representational power of the model compared with the LSTM and DNC, as the predictions are only influenced by the immediately preceding time step. Ultimately, there is a reduction in the RMS prediction error of ∼19.5% and of MA error of ∼16.1% between the LSTM and RNN models. Similarly, for the DNC, a reduction in RMS error of ∼21.0% and MA error of ∼9.7% is observed.

**TABLE 2:** Comparison of simple RNN, LSTM and DNC dynamics model RMS and mean absolute (MA) error on the validation and testing dataset, for prediction of the modified state Δx_t_ to the horizon H = 10, with required training time per epoch and inference time for a single time step.

Model	Train RMS Err	RMS Err	MA Err	Train time/epoch (s)	Infer. time (ms)
RNN	0.0899	0.5970	0.5131	97.36	2.83
LSTM	0.0507	0.4806	0.4304	118.2	3.79
DNC	0.0676	0.3796	0.3884	3644	42.8

Comparing the training and validation errors of the architectures in Table 2, a similar pattern is recorded. In practice, the validation error will be higher than the training error, as the model predictions are conditioned on the training dataset. Although the validation and test error is considerably larger than the training error, the test dataset is constituted of data collected from application of the MPC framework to a path following task, rather than the inverse damping controller. Hence, a wider range of states and actions are expected to be sampled which are unseen during the data collection process, and thus the validation and test set errors are more representative of the model performance in a real application. However, there is still good agreement between the predicted and ground truth for the majority of data points as shown in Figure 6. This demonstrates the applicability of the model to materials with unseen properties.

### 3.2 Model Predictive Control Validation

For validation of the MPC approach with visual feedback, we construct an example scenario with a model of a Nissan Leaf 2011 battery module as a sample workpiece. The module is fixed in position on a flat surface oriented parallel to the z-axis of the robot base frame, reflecting a real-world scenario where the module is extracted and secured into a fixed and approximately known position for separation of the module cover. To allow comparison with the inverse damping controller, which we refer to as the baseline, four test cases were established for surface tracking tasks over surfaces with different material properties that were not encountered in the training dataset. In each test case, a path along the surface is manually specified, but chosen differently to sample different areas of the part surface geometry. A single trial of the task is then carried out using the MPC approach, followed by two trials with the baseline controller under differing conditions. Under the former set of conditions, the baseline controller is manually tuned to best accomplish the task, while in the latter, the controller gains are manually tuned for tracking along a uniaxial path over a surface with a fifth set of environment properties. This is then applied directly to the other four test cases to establish a scenario where the controller gains are sub-optimal for each task. This encodes the essential comparison of how well the MPC controller is able to generalize to cases where the contact dynamics are changing between different tasks and materials relative to the well-tuned baseline. For each task, the angular component of the controller stiffness $K_{p}$ was held constant.

**FIGURE 7:**
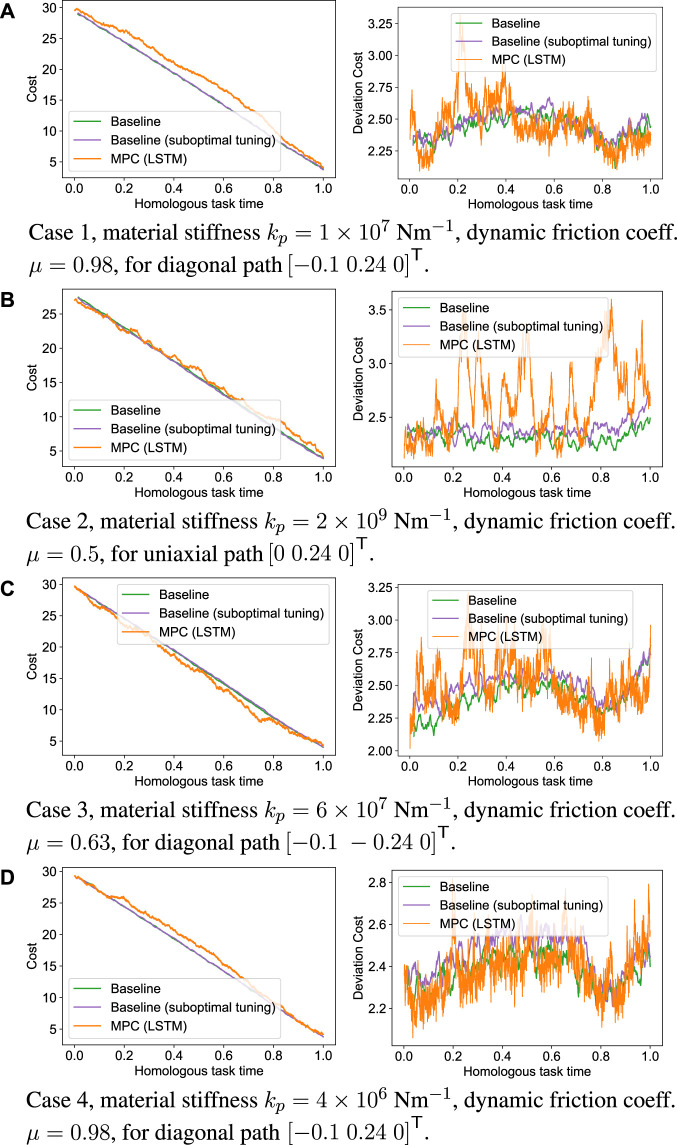
Comparison of overall cost and deviation cost components from manually tuned damping control and MPC approach. **(A)** Case 1, material stiffness k_p_ = 1 × 10^7^ Nm^−1^, dynamic friction coeff. µ = 0.98, for diagonal path (−0.1 0.24 0)^T^. **(B)** Case 2, material stiffness k_p_ = 2 × 10^9^ Nm^−1^, dynamic friction coeff. µ = 0.5, for uniaxial path (0 0.24 0)^T^. **(C)** Case 3, material stiffness k_p_ = 6 × 10^7^ Nm^−1^, dynamic friction coeff. µ = 0.63, for diagonal path (−0.1 − 0.24 0)^T^. **(D)** Case 4, material stiffness k_p_ = 4 × 10^6^ Nm^−1^, dynamic friction coeff. µ = 0.98, for diagonal path (−0.1 0.24 0)^T^.

The success criterion for each trial was achieved when the end-effector position reached within a predefined pose threshold of 10 mm from the path endpoint position $p_{n,2}$. For each trial, the cost function over the trial duration is recorded. The cost function provides several metrics that evaluate the controller performance at accomplishing the task, based on quantities such as the deviation from the desired path and path progression over time. Due to the stochastic nature of the random shooting method, the cost function has a high degree of variability over time. Hence, the 20-point moving average of the cost function was taken over each time step for the comparison. We present the results of the validation cases in Figure 7.

For all of the tasks presented, both the MPC and manually tuned baseline controllers were able to meet the success criterion and finish the planned path. The exceptional case 2 (Figure 7B) was for the trial on the material with the highest stiffness. In this case, although the MPC approach was able to effectively track the module surface and converge toward the path endpoint, large increases in the deviation cost were observed over specific intervals. These deviations arise due to inaccuracies in the learned contact model. For the latter case, the material stiffness was beyond the range encountered in the training dataset, which results in an extrapolation of the learned dynamic model. This could suggest the original training dataset was inadequate for this case, and that iterative improvement of the model is required for more challenging tasks, as suggested in Williams et al. (2017). For the collected training dataset, a probable cause is that only a small subset of the potential action space is sampled based on the deterministic damping control law, in contrast to the stochastic forward shooting method used.

Considering the other three test cases (Figures 7A,C,D), when considering the deviation from the desired path, encoded in the deviation component of the cost function, our control framework was able to track the desired path with similar accuracy to the well-tuned baseline without changes to accommodate the specific task. However, notable with the MPC approach is that the variance of the cost, or path deviation is considerably higher than for the baseline approach. This is surmised to be due to a combination of factors: the foremost being the limited number of random trajectories that can be simulated at each update step of the controller. Furthermore, as the optimization problem in (Eq. 13) is solved in three dimensions (corresponding to the three position degrees of freedom), the size of the solution space is greatly increased. Therefore at each time step, (Eq. 13) is not solved to optimality, but rather for the point in the action space sampled closest to the optimum. Further contributing factors are the slower update rate of 50 Hz relative to the baseline, and the error between predicted and ground truth displacements of the learned dynamic model. When the MPC control law is evaluated, roll-outs are computed based on this model. If the model is slightly inaccurate, the roll-outs will also be slightly inaccurate, hence the action that results in the lowest estimated cost function will not, in principle be the same as the lowest *true* cost function.

**FIGURE 8:**
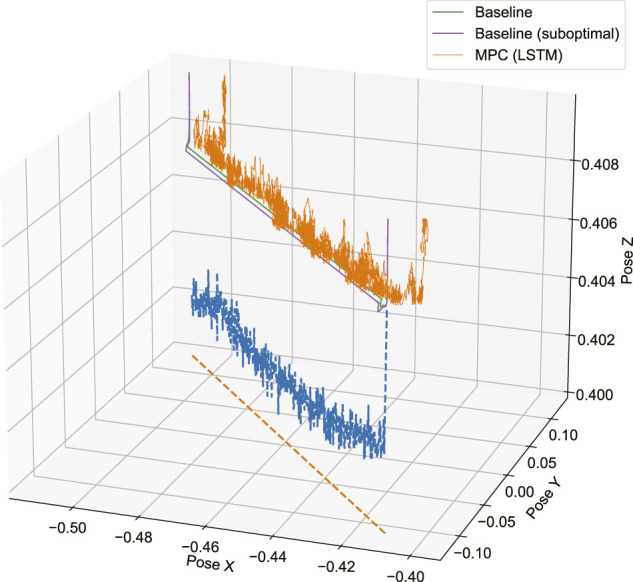
3D perspective view of the tool path in case 4 (Figure 7) under inverse damping control with manual tuning for task (green), baseline with no specific tuning for material (purple), and MPC controller with LSTM dynamic model (orange). Shown for comparison is the original planned path (orange dashed line), and the online modified reference path (blue dashed line).

Figure 8 shows a 3D representation of the planned path and vision modified reference path, comparing the baseline with specific tuning for material, baseline with no specific tuning and MPC with LSTM dynamic model. A demonstrative video of the comparison of these trials is available in Video 1 of the Supplementary Material. Here, the baseline with no specific tuning is shown to deviate from the desired path near the beginning of the trajectory, and the controller is unable to correct for this constant path deviation. Without online modification of the reference path, inaccuracies in the planned path with respect to the surface geometry lead to excessive normal forces when contact is initiated with the environment. This results in deviations from the path as the tool slides laterally along the workpiece surface. With manual tuning for the task, the controller is able to maintain the desired path as the controller is sufficiently compliant such that the contact forces are limited. In both of the baseline examples, the tool is driven toward the surface with a fixed rate reference trajectory following the desired path. With the learning-based MPC approach, the tool trajectory is not specified, but manifests from the action solution at each time step. Instead of advancing toward the surface at a fixed rate, the controller exhibits a slow and conservative approach strategy, while the normal force is modulated through the learned relation between action and force feedback. Hence when contact is established, there is no immediate deviation from the path, and the learning-based controller is similar to the baseline if it is manually tuned for the specific material. During the cutting stage, the tool intermittently deviates from the reference path due to the aforementioned inaccuracies in the dynamic model and the size of the solution space for the action sequence at each time step. However, the learning-based MPC is able to correct for this condition in contrast to the generic baseline.

### 3.3 Future Work

A principal issue with the DNC over the LSTM is the slow inference times, which are approximately ten times slower than their LSTM counterpart. The inference time strongly scales with the size of the external memory, which has been noted as a consequence of expensive matrix operations on the memory as part of the network’s attention mechanisms. This has led to more recent iterations on MANN architecture being proposed, such as the “sparse DNC” (SDNC) (Rae et al., 2016). The LSTM model employed uses a highly optimized implementation in the CUDNN library, while there is currently no equivalent implementation for the DNC. Furthermore, the autoregressive nature of the predictive models is a notable performance bottleneck, since it is necessarily a serial operation. By employing a similar approach to that presented in works such as Nagabandi et al. (2018), the required policy can instead be learned using our MPC method to supervise training of a second DNC model. Since this removes the constraint on time allowed to compute the optimal action using the MPC method, this would allow the solution of (Eq. 13) to (or close to) optimality. This is beyond the scope of this work as the primary focus is to encode the comparison of the LSTM and DNC architectures as models of the contact dynamics as a proof-of-concept.

Besides optimization of the DNC approach, a clear future extension of this work is transferring of the low-level knowledge gained in simulation to real-world cutting trials. As the dynamics of the cutting process in the real world are more complex than considered in the simulation approach in this work, and dependent on a wider range of conditions, sim-to-real transfer must be accomplished through real-world data collection and retraining of the dynamics model. In this case, the comparison between the LSTM and DNC models would be further enhanced by the additional comparison this application presents.

## 4 Conclusion

We presented a novel control approach applicable to the problem of mechanical cutting of objects based on a model predictive control framework with a memory-augmented neural network (MANN) dynamics model, incorporating a vision-based path planning system with dynamic environment position estimation. To our knowledge, this is the first study investigating the use of MANNs in a robotic control context. Results show with our path planning framework there is a capability for cutting objects of arbitrary dimensions, while the neural network dynamic model is able to generalize to different, unseen contact dynamics between tasks. Comparison between simple RNN, LSTM and the differentiable neural computer (DNC) MANN demonstrate the DNC has superior generalizing power to data collected from materials unseen during training, with ∼21% lower RMS prediction error compared of LSTM with an equivalent network architecture. Our control framework was validated in simulation considering a range of four surface-tracking tasks for a Nissan Leaf 2011 battery module, showing the ability to outperform the baseline controller used for data collection if the best controller gains are unknown. Although all tasks were completed successfully, demonstrating the robustness of our framework to parametric uncertainties, there are issues with extrapolation of the model to materials of stiffness beyond the range of the training dataset. This results in deviations from the desired path. Furthermore, the increased inference time of the DNC model—a factor of ten greater than the LSTM—presents a significant challenge when scaling to real-time applications, necessitating further optimisations. Future work will focus on validating the control framework over real-world cutting trials, with an emphasis on transferring the knowledge gained in simulation to expedite the process of training on real materials. Performance optimization of the DNC in our control framework may be considered using more recent developments such as the sparse DNC (SDNC).

## Data Availability

The datasets presented in this study can be found in online repositories. The names of the repository/repositories and accession number(s) can be found in the article/Supplementary Material. The raw dataset used for the model training and validation and the raw data used to produce the graphs in Figure 7 can be found here: CrossRef Full Text.
